# A Study of the Influence of the HCl Concentration on the Composition and Structure of (Hydroxy)Arylsiloxanes from the Hydrolysis–Condensation Reaction of Aryltrichlorosilanes

**DOI:** 10.3390/molecules24224195

**Published:** 2019-11-19

**Authors:** Irina M. Petrova, Yury I. Lyakhovetsky, Vladimir V. Chernyshev, Nikolai S. Ikonnikov, Nataliya N. Makarova

**Affiliations:** 1 A. N. Nesmeyanov Institute of Organoelement Compounds of the Russian Academy of Sciences, 28 Vavilov St., V-334, 119991 Moscow, Russia; impetr2013@yandex.ru (I.M.P.); yulyakh@ineos.ac.ru (Y.I.L.); ikonns@ineos.ac.ru (N.S.I.); 2 Department of Chemistry of the M.V. Lomonosov Moscow State University, GSP-1, 1 Leninskie Gory, 119991 Moscow, Russia; vladimir@struct.chem.msu.ru; 3 A. N. Frumkin Institute of Physical Chemistry and Electrochemistry of the Russian Academy of Sciences, 31 Leninsky prospect, 119071 Moscow, Russia

**Keywords:** hydrolysis–condensation reactions, aryltrichlorosilanes, linear and cyclic hydroxyarylsiloxanes, NMR, APCI-MS, XRPD

## Abstract

The hydrolysis–condensation reactions of *m*-tolyl, *m*-chlorophenyl, and *α*-naphtyl-trichlorsilanes, (**1**, **2**, and **3**, respectively) in water-acetone solutions were examined for how they were influenced by the change in the concentration of HCl (C_HCl_). The composition of the products was monitored by ^29^Si NMR spectroscopy and atmospheric pressure chemical ionization mass spectrometry (APCI-MS). The acidity of the medium was shown to affect the yields of the products, and so, what products were formed. For **3**, e.g., APCI-MS showed peaks of *α*-naphtyl-T_8_ and *α*-naphtyl-T_10_ as the most abundant in the spectra taken after 48 and 240 h for the reaction conducted at C_HCl_ = 0.037 mol L^−1^. Unlike this, at C_HCl_ = 0.15 mol L^−1^, those peaks were of [*α*-naphtyl(HO)_2_SiO]_2_(*α*-naphtyl)(HO)Si and/or [*α*-naphtyl(HO)Si]_3_, [*α*-naphtyl(HO)Si]_4,5_, and *α*-naphtyl-T_8_ after 192 h. However, at both C_HCl_ values, the main product (and an intermediate) after 24 h was *trans*-1,1,3,3-tetrahydroxy-1,3-di-*α*-naphtyldisiloxane. It was isolated and its structure established by ^1^H-, ^29^Si-NMR, and X-ray powder diffraction.

## 1. Introduction

Organosilicon chemistry is a developing area of chemistry. Organosilsesquioxanes are probably among the most important compounds within the whole family of organosilicon derivatives. Polyhedral oligomeric silsesquioxanes with mono and multifunctional groups or with organic groups of different structures are important building blocks for the obtainment of electroluminescent, electrochromic, organic optoelectronic materials, and molecules capable of tunable surface reactions. They are also used as building blocks for hybrid nanocomposites because of their nanometric size [1,2,3,4]. Polyorganosilsesquioxanes (POSSO) can be attributed to hybrid polymeric materials in which macromolecules with an inorganic siloxane backbone are framed by side organic groups R [5]. Depending on the order of the T-linking, polymer molecules with different stereoregularity of the cyclolinear chain, polyhedral framework molecules with different degrees of completeness, and dendrimers and polymers with irregular branched structures are formed. More than 50 years ago, a high molecular weight, soluble polyphenylsilsesquioxane (PPhSSO) possessing valuable properties for practical use was obtained [6].

The synthesis of PPhSSO initiated the obtainment of polyorganosilsesquioxanes (POSSO) with different organic groups at the silicon atoms and the study of their structure and properties [5]. It was previously shown that depending on the conditions of condensation and polymerization, products with different structures, including polyhedral compounds, were formed [7]. A detailed study of the hydrolysis–condensation reactions of cyclohexyl and phenyltrichlorosilane was carried out to elucidate the influence of the reaction conditions upon the composition of products formed as incomplete and completed cage-like structures [8,9]. In the case of organotrichlorosilanes, incomplete polyhedral organosilsesquioxanes with cyclic alkane substituents were purposefully synthesized by controlling the reaction conditions [10,11,12,13]. Also, the hydrolysis-condensation reaction of methoxy(3-oxyethylene)propyltrichlorosilanes with different numbers of oxyethylenes at different concentrations in solution and pH values was investigated [14]. Depending on the conditions, trihydroxyorganosilanes or tetrahydroxydiorganodisiloxanes were the main products of the reaction.

The hydrolysis and self-condensation reaction of methyl(trimethoxy)silane in the presence of an acid as a catalyst was studied by ^29^Si NMR spectroscopy, but the formation of the first compounds with the [Me(OR)_2_Si]_2_O and MeSi(OR)[OMeSi(OR)_2_]_2_ bonds was only monitored [15]. The method was used in the study of alterations in the composition of primary intermediates in the hydrolysis–condensation reaction of phenyltrichlorosilane leading to (tetrahydroxy)(tetraphenyl)cyclotetrasiloxane, [PhSi(HO)O]_4_ [16]. The ^29^Si NMR spectroscopy method demonstrated that the reaction of hydrolysis–condensation of phenyltrimethoxysilane in the presence of a basic catalyst in different conditions occurred through an intermediate, tetrahydroxy(diphenyl)disiloxane [17]. When the reaction mixture was diluted, dodecaphenyldodecasilsesquioxane (phenyl-T_12_) was obtained in 95% yield, while an increase in the concentration of the starting compound led to PPhSSO [17,18]. Hydrolysis of HSi(OMe)_3_ in an acidic medium gave rise to tetramethoxydisiloxane as an intermediate compound, the thermal polycondensation of which afforded ladder organosilsesquioxane [19]. The step by step hydrolysis of phenyltrimethoxysilane in the presence of an acid gave tetrahydroxy(diphenyl)disiloxane. It was used in the reaction of polycondensation that produced stereoregular PPhSSO [20]. Recently, the condensation reaction of PhSi(HO)_3_ in the presence of different acids was studied by high performance liquid chromatography (HPLC) and ^29^Si NMR spectroscopy. Tetrahydroxy(diphenyl)disiloxane was found to be the main product rather than the linear trisiloxane. The further condensation led to the formation of cyclotri- (two isomers) and cyclotetrasiloxanes [21]. Thus, tetrahydroxy(diphenyl)disiloxane proved to be a block for forming more complex organosilicon oligomers and polymers. Interesting results were obtained for different organotrichlorosilanes when they were transformed to sodium organocyclosiloxanolates and acetic acid was added. [*i*-PrSi(HO)O]_4_ was isolated with a 93% yield, while [*p*-BrPhSi(HO)O]_4_ was not formed [22]. These results indicate that the instability of hydroxyorganosiloxanes depends on the nature of the substituent at the silicon atoms [22].

Hydrolysis or hydrolysis–condensation of xylyl-, *m*-tolyl-, and *β*-naphtyltrimethoxysilanes was performed in self-assembled, hollow compounds, Pd ^II^ or Pt^II^ coordination hosts (cage, bowl, or tube) in D_2_O. The results strongly depended on the host type and the organic substituent at the silicon atom. Triols, dimer siloxanes, and cyclotrisiloxanes were obtained and isolated as encapsulated in supramolecular complexes [23,24,25,26]. The hydrolysis of organotrialkoxysilanes with stoichiometric amounts of sodium hydroxide or potassium led to the formation of organocyclosiloxanolates. However, in the presence of metal salts, frame metallosiloxanes formed. The treatment of these compounds with HCl gave rise to stereoregular organocyclosiloxane [27,28,29].

Another study of the similar hydrolysis, this time of phenyltrimethoxysilane, was carried out with stoichiometric amounts of sodium hydroxide or sodium metal in the presence of salts of transition and non-transition metals. Sandwich and frame metallosiloxanes with one and two metals were obtained [30,31,32,33,34].

The synthesis of isomers of organocyclosiloxanes by different methods is of interest not only because they can be initial blocks for the obtainment of stereoregular cyclolinear polyorganosilsesquioxanes [35], but these isomers can also be modified by the addition of side mesogenic groups to give isomeric liquid crystal (LC) compounds. This provides possibility to study the influence of the stereoisomeric structures of these species on the type of packing in the LC state [36].

All of this demonstrates that in spite of the fact that there have been publications on the hydrolysis of organotrichloro-, organotrialkoxysilanes and the condensation of their hydrolysates in the presence of acids and bases in different solvents or in aqueous solution, the mechanism of the process is not yet properly defined. It is especially because of the insufficient information on the structures of intermediates in these reactions. Our previous study of the polycondensation of (tetrahydroxy)(tetraaryl)cyclotetrasiloxanes showed that the polycondensation in the presence of montmorillonite produced polymers with a greater *M_w_* than in the case of its absence. It also showed mass spectrometry to be a good tool for detecting intermediates and for their identification [37]. It is clear and comes from the above that the results of the hydrolysis–condensation reactions of organotrichlorosilanes should depend on the reaction conditions, and in particular, on the acidity of the reaction mixture. Taking this in account we undertook a study of the hydrolysis-condensation reactions of three organotrichlorosilanes conducted at different acidities of the reaction mixtures, monitoring the processes with positive ion mode atmospheric pressure chemical ionization mass spectrometry (PI APCI-MS) and ^29^Si-NMR spectroscopy.

## 2. Results and Discussion

**Figure 1:**
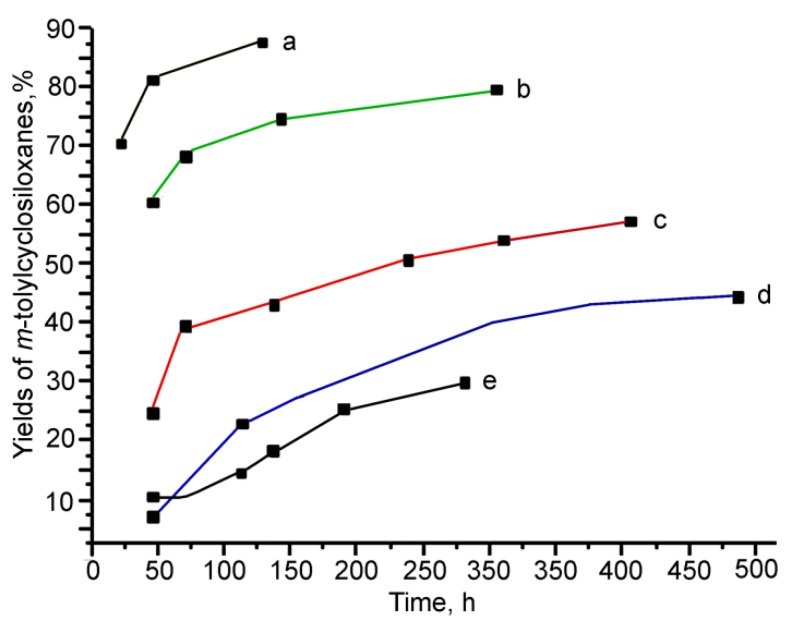
Dependence of the yields of *m*-tolylcyclosiloxanes from the hydrolysis-condensation reaction of **1** on time at different concentrations C_HCl_ in water-acetone solutions: curve **a**, C_HCl_ = 0.40; **b**—0.15; **c**—0.076; **d**—0.054; and **e**—0.038 mol L^−1^.

As aforesaid, the article deals with three substituted aryltrichlorosilanes, *m*-tolyl-, *m*-chlorophenyl- and *α*-naphthyltrichlorosilanes (**1**, **2**, and **3**, respectively), and their hydrolysis–condensation reactions in water-acetone media at different dilutions, and thus, different acidities of the medium. These organotrichlosilanes were chosen for the study since **1** and **2** had the substituents at the silicon atoms with close Van der Waals volumes, with different electronic properties. The substituent in compound **3** possesses the larger Van der Waals volume. From all of this, it could be anticipated that products of their reactions would be different. The reactions were conducted with the concentrations of HCl (C_HCl_) between 0.032 and 0.40 mol L^−1^ at 4 °C, and the yields of the products were determined in the interval of 24–720 h (see Note 1 in the Supplementary Materials (SM)). Figure 1 shows these yields of the products from the reaction of **1** that precipitated from the solution depending on the reaction time (see Note 2 in the SM). As can be seen from Figure 1, the rate of the condensation of **1** and final yields of the products decreased with the decrease in acidity.

**Figure 2:**
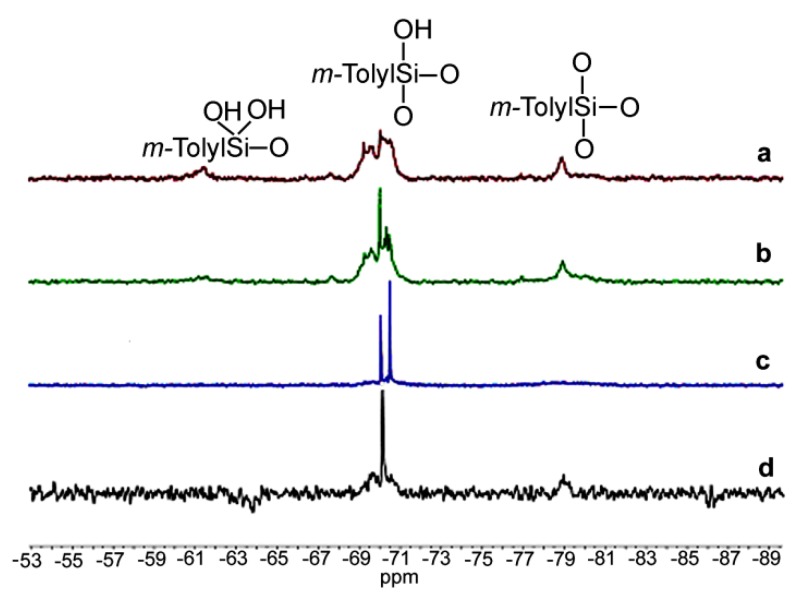
^29^Si NMR spectra of *m*-tolylcyclosiloxane compounds from the reaction of **1** carried out for 48 h at C_HCl_ = 0.40 (**a**), 0.15 (**b**), 0.054 (**c**), and 0.038 mol L^−1^ (**d**).

Figure 2 presents the ^29^Si NMR spectra of *m*-tolylcyclosiloxane compounds obtained at C_HCl_ = 0.40, 0.15, 0.054, and 0.038 mol L^−1^, separated from the water-acetone solution after 48 h, and dissolved in acetone-d_6_.

Three groups of signals were observed in these spectra of the products obtained at C_HCl_ = 0.40 and 0.15 mol L^−1^: at −61.0 to −62.0; −69.3 to −71.3; and −78.5 to −79.5 ppm. They coincided very closely with those from the ^29^Si NMR spectra of phenylsiloxane products obtained by the hydrolysis-condensation of PhSiCl_3_ at C_HCl_ = 0.35 mol L^−1^ [16]. Based on the results of this study, we attributed the above signals to compounds with two OH groups at the silicon atoms, one group, and no groups, respectively (see Note 3 in the SM). In the ^29^Si NMR spectra of *m*-tolylcyclosiloxane products obtained at C_HCl_ = 0.054 and 0.038 mol L^−1^, two singlets at −70.05 and −70.43 ppm, and weak signals in the region of −78.5 to −79.5 ppm were present, while no signals in the region of −61.0 to −62.0 ppm were detected. In general, all spectra evidenced that the main products precipitated from the reaction mixtures up to 48 h were those, most, if not all of which, were of the second type; namely, containing one free OH group at each silicon atom.

**Figure 3:**
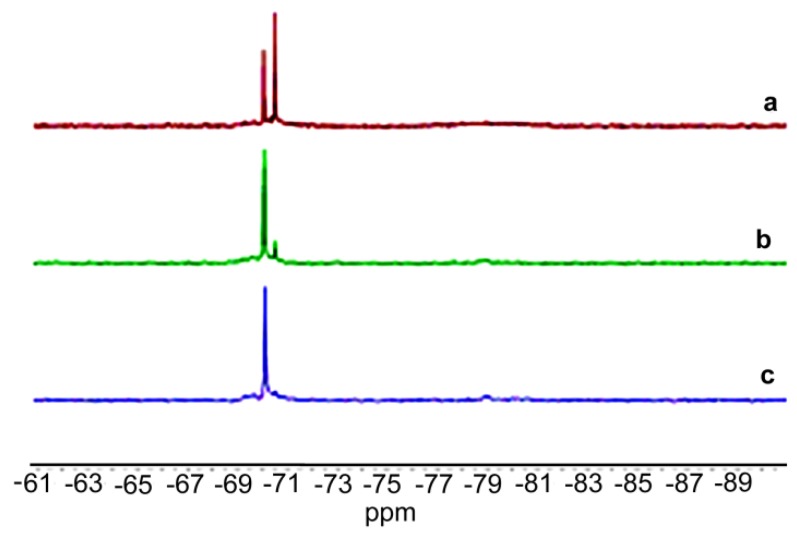
^29^Si NMR spectra of *m*-tolylcyclosiloxanes obtained from the reactions of **1** conducted at C_HCl_ = 0.054 mol L^−1^ for 48 h (**a**), 120 h (**b**), and 475 h (**c**).

Figure 3 presents the dynamics of the ^29^Si NMR spectra of *m*-tolylcyclosiloxane products separated from a water-acetone solution for the reaction of **1** conducted at C_HCl_ = 0.054 mol L^−1^. In the spectrum recorded after 48 h, as already mentioned, the singlets were registered at −70.05 and −70.43 ppm. According to [35,36,37], they had to belong to all *cis*-(tetrahydroxy)(tetra-*m*-tolyl)tetracyclosiloxane (**4,** downfield signal) and the *cis-trans-cis*-isomer (upfield signal). In the course of the further reaction, the upfield signal decreased and almost completely vanished after 475 h. This indicated that the *cis-trans-cis*-isomer likely graded into the all *cis*-isomer **4** in the course of the reaction. However, an alternative yet exists: if the *cis*-*trans*-*cis*-isomer was less soluble than all *cis*-isomer, it was probably deposited from the reaction mixture to a significantly greater extent than the latter to the point of its absence in the mixture, and thus, in the precipitate. The signals in the region of −78.5 to −79.5 ppm were virtually absent. All of this evidenced that the polycondensation did not take place to any significant extent and the final product was the abovementioned all-*cis*-isomer **4**.

The above results were in good accordance with those of TLC in a mixture of toluene:ether = 1.0:0.75 as an eluent. Two spots with R_f_ = 0.05 and 0.50 were observed for the 48 and 120 h products, while only a single spot with R_f_ = 0.05 was for the 475 h product.

**Figure 4:**
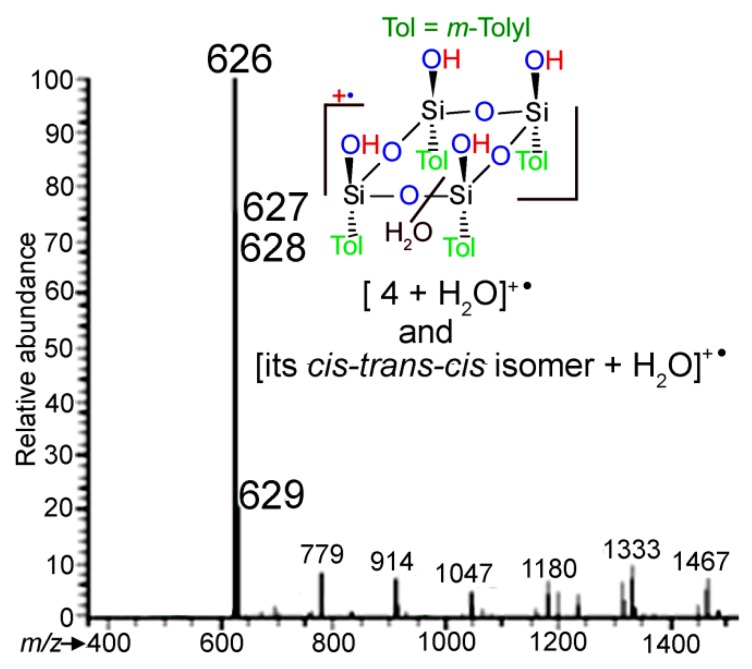
PI APCI mass spectrum of the precipitate separated from the reaction mixture after the hydrolysis–conduction reaction of **1** was carried out at C_HCl_ = 0.054 mol L^−1^ for 120 h.

Another piece of support came from the PI APCI mass spectrum of the precipitate separated from the reaction mixture after 120 h that displayed the hydrates of the molecular ions of **4** and, apparently, its *cis-trans-cis*-isomer at *m*/*z* 626 (Figure 4). Water obviously added in the mass spectrometer, as shown by the previously recorded mass spectra of similar compounds [37] and by the ^29^Si NMR spectrum (Figure 3b), attesting to the lack of the second OH group at the silicon atoms (see Note 4 in the SM). The mass spectra of the precipitates separated from the 48 and 475 h reaction mixtures were similar.

This method was also used to determine the composition of products of the hydrolysis–condensation reaction of silane **1** performed at C_HCl_ = 0.40 mol L^−1^ (Note 5 in the SM).

**Figure 5:**
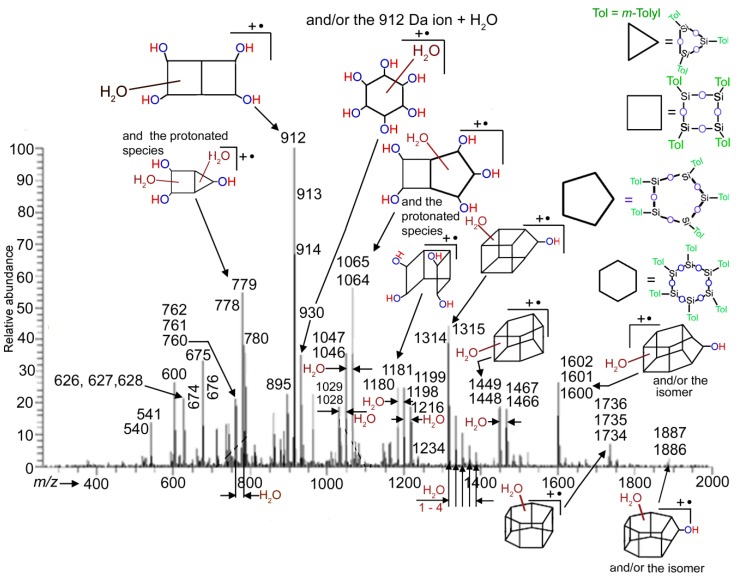
PI APCI mass spectrum of a mixture of *m*-tolylcyclosiloxane products from the hydrolysis-condensation reaction of **1** conducted at C_HCl_ = 0.40 mol L^−1^ for 24 h (see Note 6 in the SM).

**Figure 6:**
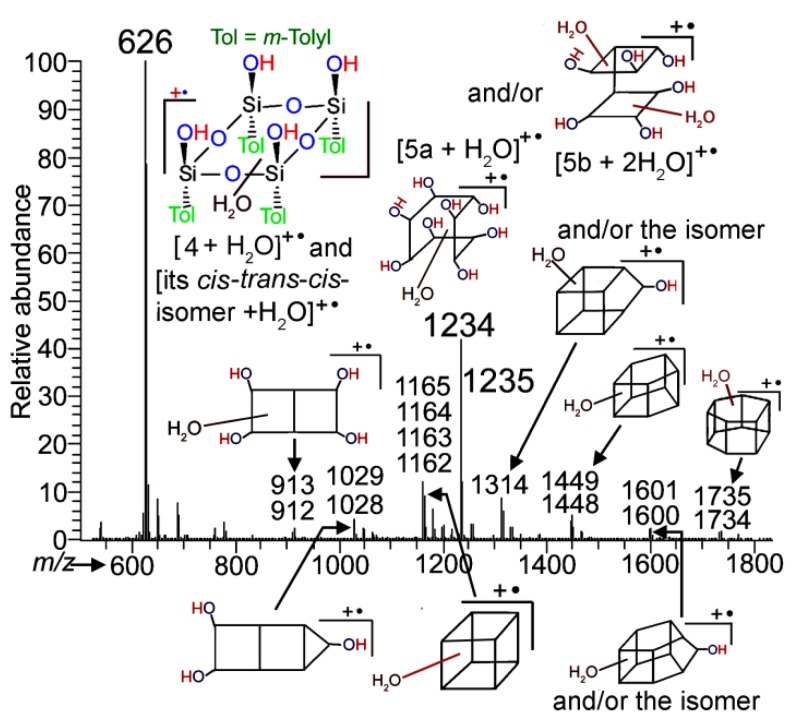
PI APCI mass spectrum of a mixture of products from the hydrolysis–condensation reaction of compound **1** conducted in a water-acetone solution at C_HCl_ = 0.40 mol L^−1^ for 48 h.

Figure 5 depicts the PI APCI mass spectrum of a mixture of hydrolysis-condensation products of compound **1** from the reaction conducted at C_HCl_ = 0.40 mol L^−1^ for 24 h. A great number of peaks in the mass spectrum show that the products of the hydrolysis-condensation reaction of **1** included hydroxy(*m*-tolyl)cyclosiloxanes with a single tetracyclosiloxane ring, with double fused rings, and also polyhedral structures with different completeness. When the reaction was conducted for 48 h, the mass spectrum changed drastically. The main products turned out to be compound **4** and its *cis*-*trans*-*cis*-isomer registered as radical cations of their hydrates with the nominal mass of 626 Da, and [*m*-tolyl(OH)SiO]_8_ (**5a**) and/or {[*m*-tolyl(OH)SiO]_3_-*m*-tolylSiO_1.5_}_2_ (**5b**) were also detected as a hydrate and a dihydrate, respectively, both with the 1234 Da nominal mass. The peaks of all other products were of minor abundances (Figure 6).

A similar spectrum was recorded for the precipitate obtained after 120 h in which the relative abundance of the peaks of **5** increased pronouncedly, however (Figure S1 in the SM).

When the concentration of HCl was reduced to 0.15 mol L^−1^, the mass spectrum recorded after 48 h from the beginning of the reaction showed an abundant peak of compound **4** and its isomer, the peaks of other products being significantly less abundant or even of minor abundances (Figure S2 in the SM).

**Scheme 1:**
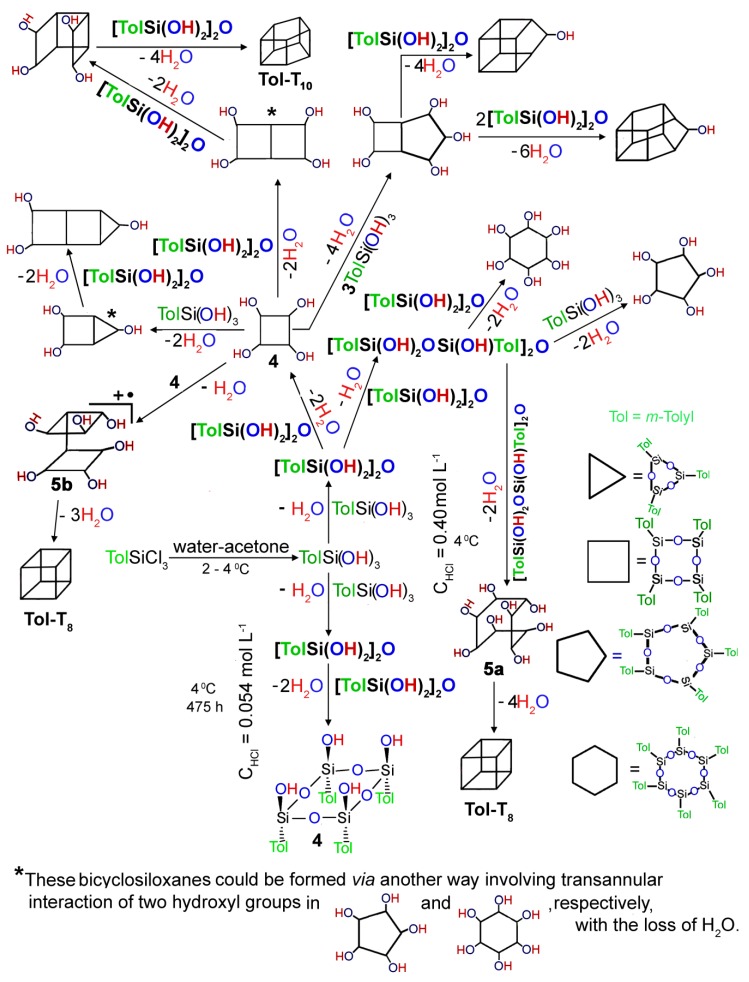
The hydrolysis-condensation reaction of *m*-tolylSiCl_3_ (**1**) catalyzed by HCl formed via the hydrolysis of compound **1**.

On the basis of the PI APCI mass and ^29^Si NMR spectra of *m*-tolylcyclosiloxane products, it is possible to present Scheme 1 of the hydrolysis-condensation reaction of *m*-tolylSiCl_3_ (**1**) in water–acetone media. The scheme displays the formation of the main products and intermediates. For other products and intermediates and the mass numbers of all species detected, see Figure 5, Figure 6, Figures S1 and S2.

The precipitate obtained from the reaction at C_HCl_ = 0.054 mol L^−1^ up to and including the 475 h time point was recrystallized from diethyl ether to furnish compound **4** in 30.8% yield.

**Figure 7:**
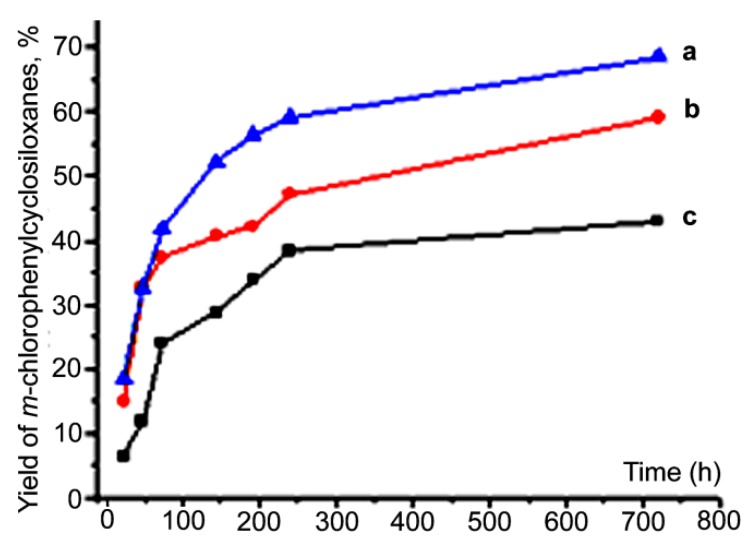
Dependence of the yield of *m*-chlorophenylcyclosiloxanes from the hydrolysis–condensation reaction of **2** on time at different concentrations C_HCl_ in water-acetone solutions: 0.056 (**a**), 0.047 (**b**), and 0.032 mol L^−1^ (**c**).

The hydrolysis–condensation reaction of *m*-chlorophenyltrichlorosilane (**2**) was carried out at 4 °C in water-acetone solutions at C_HCl_ = 0.032–0.056 mol L^−1^. Figure 7 presents the yields of reaction products as a function of time. It shows that the final yield of the products increased with the increase in C_HCl_.

**Figure 8:**
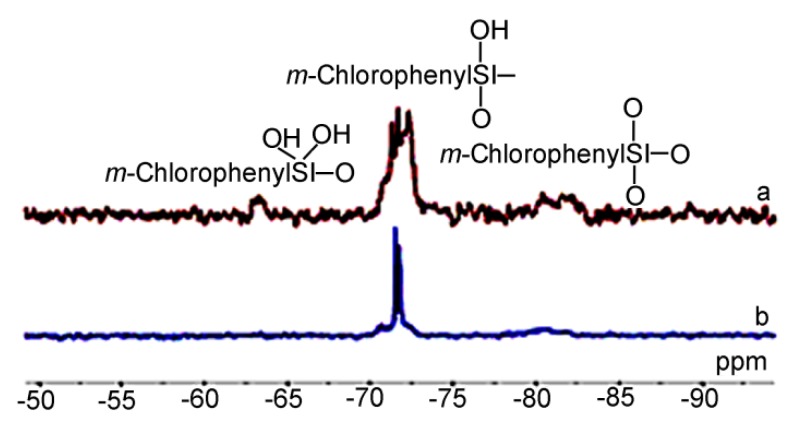
^29^Si NMR spectra of *m*-chlorophenylcyclosiloxane compounds obtained from the hydrolysis-condensation reaction of **2** conducted at C_HCl_ = 0.056 (**a**) and 0.032 (**b**) mol L^−1^ for 48 h.

Figure 8 gives the ^29^Si NMR spectra of the precipitates taken after 48 h from the beginning of the reaction (see Note 7 in the SM).

In contrast to the ^29^Si NMR spectrum of *m*-tolylcyclosiloxane compounds obtained at C_HCl_ = 0.054 mol L^−1^ after 48 h, where two singlets were observed in the range of −70.0 to −71.0 ppm (Figure 3, trace **a**), in the ^29^Si NMR spectrum of *m*-chlorophenylcyclosiloxane products obtained from the reaction at C_HCl_ = 0.056 mol L^−1^ after the same time, three groups of signals at −62.9 to −63.5, −71.3 to −72.3, and −80.0 to −82.2 ppm were observed. Meanwhile, three groups of the signals with close chemical shifts were present in *m*-tolylcyclosiloxane products obtained after 48 h at C_HCl_ = 0.15 and 0.40 mol L^−1^ (Figure 2, traces **a** and **b**). It should also be highlighted here, that in the case of C_HCl_ = 0.032 mol L^−1^, the spectrum of *m*-chlorophenylcyclosiloxane products displayed two singlets (on the background of a very small broad signal) at −71,3 to −72.3 ppm and an insignificant broad signal at −80.0 to −82.2 ppm Thus, the increase of C_HCl_ to 0.056 mol L^−1^ led to further condensation of *m*-chlorophenylsiloxane products with the formation of poly-*m*-chlorophenylsilsesquioxanes, as inferred from the appearance of the noticeable broad signal in the region −80.0 to −82.2 ppm. The signal was attributed to poly-*m*-chlorophenylsilsesquioxane with different conformational and configuration sequences of units that were inherited from the isomers of (tetrahydroxy)(tetra-*m*-chlorophenyl)cyclotetrasiloxane that manifested themselves in the ^29^Si NMR spectrum in the region of −71.3 to −72.3 ppm (Figure 8**)**.

The ^29^Si NMR spectrum of a mixture of products obtained after the 90 h reaction at C_HCl_ = 0.032 mol L^−1^ proved to be similar to that of the products from the 48 h reaction with the singlets retained in the original ratio. A similar ^29^Si NMR spectrum was obtained for the precipitate taken after 48 h for the reaction carried out at C_HCl_ = 0.047 mol L^−1^. However, the spectrum for the 72 h reaction at C_HCl_ = 0.056 mol L^−1^ displayed only the broad signal in the region of −78.5 to −81.8 ppm (Figure S3 in the SM). The comparison of the data of the ^29^Si NMR spectra in Figure 3 with the above results showed that at close values of the Van der Waals volumes of the *m*-tolyl- and *m*-chlorophenyl groups but with the different inductive effects of these substituents, the reactivities of the OH-groups of the corresponding intermediates, including hydroxyorganocyclotetrasiloxanes, in the presence of HCl, were different. It led to the formation of poly-*m*-chlorophenylsilsesquioxanes at a concentration of C_HCl_ = 0.056 mol L^−1^ in the case of the *m*-chlorophenyl derivative, while the polycondensation did not occur for *m*-tolyl-one at virtually the same concentration of the acid (C_HCl_ = 0.054 mol L^−1^).

The following should be mentioned about two singlets registered in the ^29^Si NMR spectra in the region of −71.3 to −72.3 ppm. They can be ascribed to *cis*-*trans*-*cis*-(tetrahydroxy)(tetra-*m*-chlorophenyl)cyclotetrasiloxane (**6a**) (upfield signal) and its all *cis*-isomer (**6b**) (downfield signal) according to [31,32,33]. All of this found support in the TLC data. Earlier, we made the assignments of the R_f_ values for four isomers of [Ph(OH)SiO]_4_ based on the X-ray study of their synthesized liquid crystal derivatives [36]. These TLC values were later confirmed in another publication where X-ray single crystal determination of the all *trans*-isomer was performed and three singlets characteristic of the *cis*-*cis*-*trans*-isomer were registered in the ^29^Si NMR spectrum [22]. The R_f_ of **6a** and **6b** were close to those of the corresponding phenyl analogs.

The combined precipitate from the reaction carried out at C_HCl_ = 0.056 mol L^−1^ for 240 h was recrystallized fractionally to afford isomers of (tetrahydroxy)(tetra-*m*-chlorophenyl)cyclotetrasilloxane (**6a** (24.9%) and **6b** (19.0%)) that were characterized by ^1^H and ^29^Si-NMR spectra (see Section 3.2).

**Figure 9:**
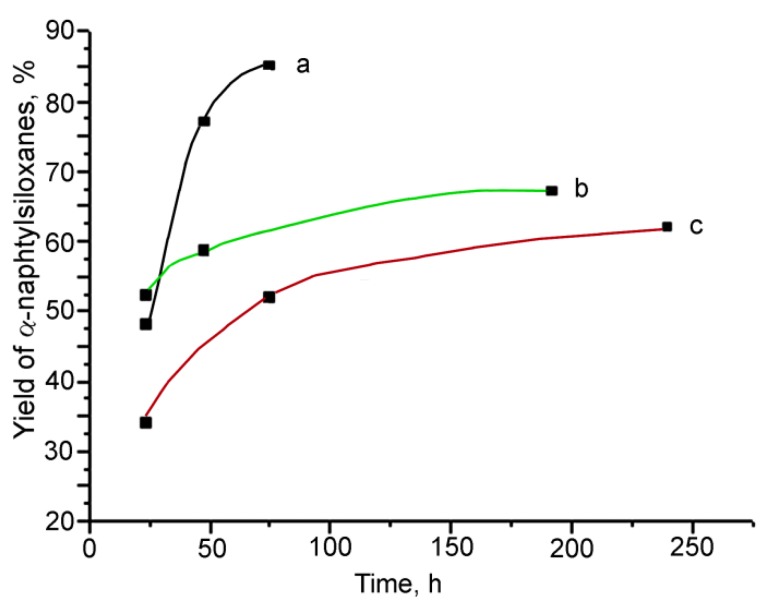
Dependence of the yields of *α*-naphthylsiloxanes from the hydrolysis–condensation reaction of compound **3** on time at different C_HCl_ in water-acetone solutions: (**a**) C_HCl_ = 0.37, (**b**) 0.15, and (**c**) 0.037 mol L^−1^.

A study of the hydrolysis–condensation reaction of *α*-naphtyltrichlorosilane (**3**) was carried out at C_HCl_ = 0.37–0.037 mol L^−1^ in water-acetone solutions at 4 °C. Figure 9 shows the yields of *α*-naphthylsiloxane products depending on C_HCl_ and the reaction time.

The maximum yields of the products proved to be at C_HCl_ = 0.37 mol L^−1^. With the decrease of C_HCl_ to a value of 0.037 mol L^−1^, the yields decreased drastically.

**Figure 10:**
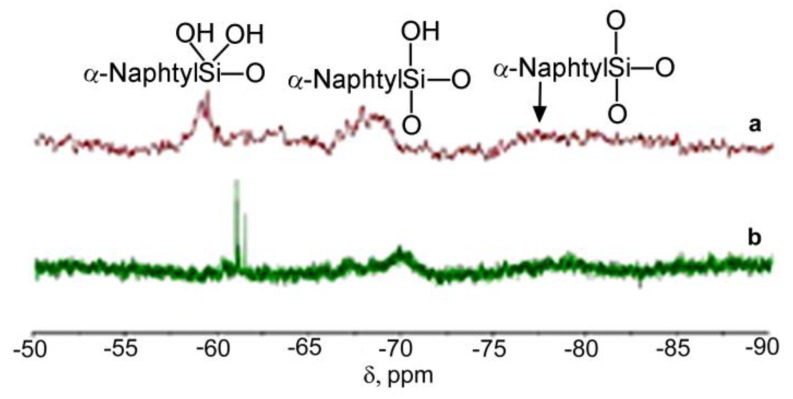
^29^Si NMR spectra of *α*-naphthyllsiloxane compounds from the reaction conducted for 48 h: (**a**) C_HCl_ = 0.37; (**b**) 0.15 mol L^−1^.

**Figure 11:**
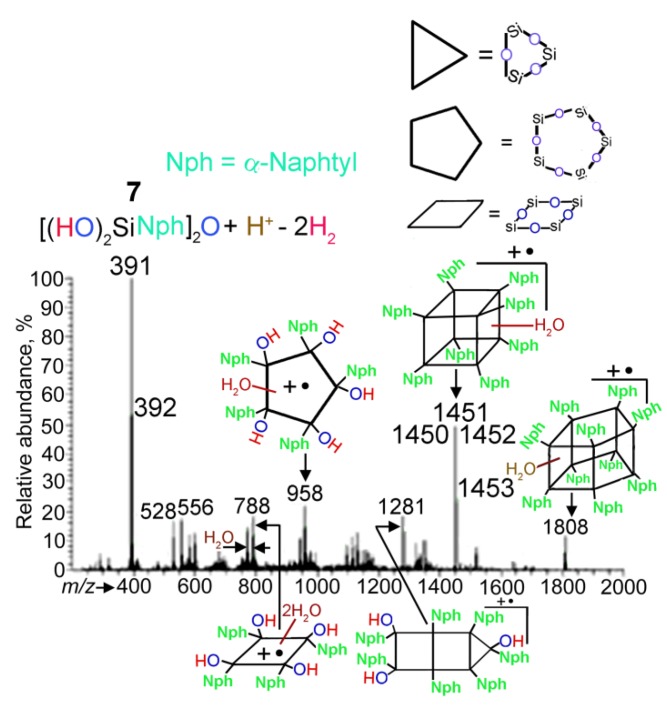
PI APCI mass spectrum of *α*-naphtylsiloxane compounds from the hydrolysis-condensation reaction of **3** carried out at C_HCl_ = 0.037 mol L^−1^ for 24 h.

Figure 10 gives the ^29^Si NMR spectra of *α*-naphtylsiloxane products obtained at C_HCl_ = 0.37 and 0.15 mol L^−1^; for C_HCl_ = 0.37 mol L^−1^, two broad signals were observed in the regions of −59.0 to −59.3 and −66.4 to −69.1 ppm (Figure 10a). This means that a great number of siloxane products with one and two hydroxyl groups at the silicon atoms were present in this time precipitate. The decrease in C_HCl_ to 0.15 mol L^−1^ led to a decrease in the rate of the condensation reaction of α-naphtyltrihydroxysilane and 1,1,3,3-tetrahydroxy-1,3-di-*α*-naphtyldisiloxane, and two singlets at −61.34 and −60.68 ppm clearly manifested themselves in the region characteristic of siloxanes containing only RSi(OH)_2_ fragments. Most likely, the singlets belonged to 1,1,3,3-tetrahydroxy-1,3-di-*α*-naphtyldisiloxane in different conformations. However, a broad signal at −69 to −71 ppm (region for products bearing one hydroxyl at each silicon atom) was detected, but with a significantly less intensity that in the case of C_HCl_ = 0.37 mol L^−1^. Interestingly, a signal of organocyclosiloxanes with no hydroxyl groups at −78.0 to −81.0 ppm was also observed but on the background level only. The same singlets were found for the case of C_HCl_ = 0.037 mol L^−1^, but in another ratio that produced a slight shift in their positions. In this case, other products in the precipitates were determined by PI APCI-MS (Figure 11 and Figure S4).

The precipitates from the reactions of **3** conducted at C_HCl_ = 0.037, 0.15, and 0.37 mol L^−1^ from several time points were isolated. Recrystallization of them from CH_3_CN gave white powders, each with m.p. 162 °C (compound **7**; see below). Compound **7** was characterized by ^1^H and ^29^Si NMR. If in the ^29^Si NMR spectra of compounds **4**, **6a**, and **b** were signals at −70.16, −71.63, and −71.89 ppm, respectively, for compound **7,** the signals shifted downfield to −60.0–−61.0 ppm.

As aforesaid, the compositions of the unrecrystallized precipitates, obtained at several time points from the reaction conducted at C_HCl_ = 0.037 mol L^−1^, were determined by the PI APCI-MS. Figure 11 presents the mass spectrum of *α*-naphthylsiloxane compounds obtained when the reaction was carried out at C_HCl_ = 0.037 mol L^−1^ for 24 h. Two main compounds were registered: 1,1,3,3-tetrahydroxy-1,3-di-*α*-naphtyldisiloxane (**7** and probably, its other conformational isomers) as its (M + H − 2H_2_)^+^ ion with the nominal mass 391 Da (see below), and *α*-naphtyl-T_8_ as the hydrate of its radical cation (nominal mass 1450 Da). Besides, small abundance peaks of ions of some other products were present in the spectrum, the monohydrate and the dihydrate of [*α*-naphthyl(OH)SiO]_4_^+•^ (nominal masses 770 and 788 Da, respectively); the hydrates of [*α*-naphthyl(OH)SiO]_5_^+•^(nominal mass 958 Da); and *α*-naphthyl-T_10_^+•^ (nominal mass 1808 Da) being among them. The PI APCI mass spectra taken for this reaction carried out for 48 and 240 h are given in the SM (Figure S4). Both spectra showed that in these precipitates the main products proved to be *α*-naphtyl-T_8_ and *α*-naphtyl-T_10_ registered again as the hydrates of their radical cations. With that, a small abundance ion peak of **7** at *m*/*z* 391 was still present in the former spectrum, while virtually absent in the latter one. The structures of other products formed in essentially less amounts and registered as the radical cations or their hydrates are depicted in the figure (Note 8 in the SM).

A similar set of mass spectra was taken for the reaction implemented at C_HCl_ = 0.15 mol L^−1^. For the precipitate obtained from the reaction conducted for 24 h, a negative ion mode (NI APCI) mass spectrum was also obtained. The spectrum displayed an abundant ion peak of radical anion of **7** at *m*/*z* 394. This finding evidenced that the 391 Da ion in the PI APCI mass spectra (Figure 11and Figure S4a) really belonged to species **7** in spite of its rather unusual type. Fragmentation of its protonated molecule with elimination of two hydrogen molecules was likely to have occurred, as an ‘in-source collision-induced dissociation’ process [38,39]. The PI APCI mass spectrum of the precipitate obtained after 192 h is depicted in the SM as Figure S5. This time, the main products turned out to be *α*-naphtyl-T_8_; cyclosiloxanes [(*α*-naphtyl)Si(OH)O]_4,5_, registered as the hydrates of their radical cations (nominal masses 1450, 958, and 788, 770 Da, respectively); linear trisiloxane (*α*-naphtyl)_3_Si_3_(OH)_5_O_2_, registered as its radical cation and the monohydrate of the latter; and/or cyclosiloxane [(*α*-naphtyl)Si(OH)O]_3_, registered as both the monohydrate and the dihydrate (nominal masses 582 and 600 Da, respectively). Some other products giving small abundance peaks were also detected.

**Figure 12:**
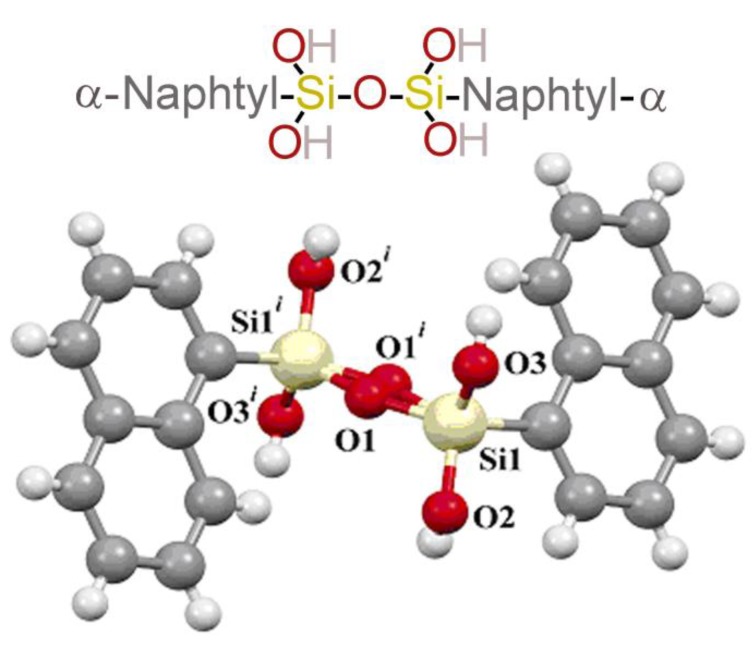
The molecular structure of **7** showing the atomic numbering of the non-carbon atoms (symmetry code (*i*) *1-x, 1-y, 1-z*). Atom O1 is disordered over two positions near the crystallographic center of symmetry at (*1/2, 1/2, 1/2*).

As mentioned above, the 24, 48, and 192 reaction time precipitates were recrystallized from acetonitrile to give a compound with a m.p. 162–164 °C (common yield 48%). The ^1^H and ^29^Si-NMR spectra (see Section 3.2) spoke in favor of the isolated compound being 1,1,3,3-tetrahydroxy-1,3-di-*α*-naphtyldisiloxane. Its structure (trans-isomer, **7**) and the type of packing were revealed by X-ray powder diffraction analysis. The crystal data, data collection, and refinement parameters are given in Table 1. The molecular structure of **7** and a portion of the crystal packing prepared with mercury [40] are shown in Figure 12 and Figure S6 (in the SM), respectively. Therein, the diffraction profile after the final, bond-restrained Rietveld refinement is shown as Figure S7.

In the crystal structure, molecule **7** is situated in the center of symmetry at (*1/2, 1/2, 1/2*). However, the molecule is not centrosymmetric because atom O1 is disordered over two positions (Figure 12) with the equal occupancies providing the Si–O–Si angle of 162.3(9)°. The Si–O bond lengths (1.534(15)–1.646(4) Å) lie within the normal ranges. The hydroxyl groups are involved in intermolecular hydrogen bonds (Table 2) which link the molecules into layers parallel to the *bc* plane (Figure S6 in the SM). The search in the Cambridge Structural Database (ConQuest, version 1.21 [41]) for close molecules resulted in only one hit with refcode *MOQMOU* [25] that contains the molecule of 1,1,3,3-tetrahydroxy-1,3-bis(2-naphthyl)disiloxane included in the framework

**Scheme 2:**
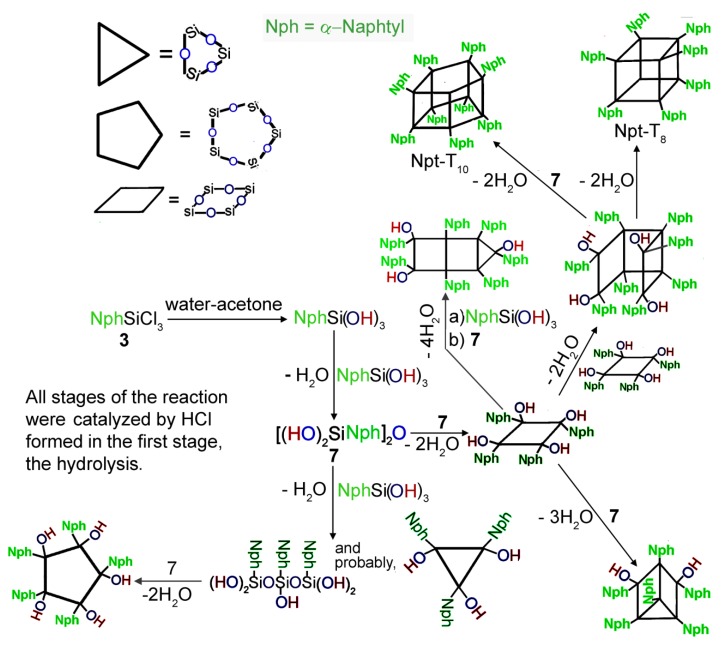
The hydrolysis-condensation reaction of compound **3.**

Based on the PI and NI APCI mass spectra, the X-ray powder diffraction data, and the ^1^H- and ^29^Si-NMR spectra of *α*-naphthylsiloxane products from the hydrolysis-condensation reaction of *α*-naphtyltriclorosilane (**3**) Scheme 2 can be suggested.

## 3. Materials and Methods

### 3.1. Materials

*m*-Tolyltriclorosilane (**1**), *m*-chlorotrichloroslane (**2**), and *α*-naphtyltrichlorisilane (**3**) were synthesized according to the protocols [42,43,44], respectively. ^29^Si NMR (CDCl_3_, δ/ppm): −0.88, −2.13, and −0.44, respectively.

Commercial grade organic solvents (acetone, acetone-d_6_, diethyl ether, benzene, and hexane) were cleaned and dried (if necessary) according to conventional procedures. Acetonitrile, acetonitrile-d_3_, and CDCl_3_ were used without additional purification.

### 3.2. Methods

The ^1^H and ^29^Si NMR spectra were taken on Bruker AV-400 and AV-600 spectrometers (Bruker Corporation, Karlsruhe, Germany) in aceton-d_6_ solutions at 20 °C.

The PI and NI APCI mass spectra were obtained with a Thermo Finnigan LCQ Advantage tandem dynamic mass spectrometer (Thermo Finnigan, San Jose, CA, USA). The instrument was equipped with an octapole ion trap mass analyzer, a Surveyor MS pump, and a nitrogen generator Schmidlin-Lab., model N2 Mistral-4 (Schmidlin-Lab, Neuheim, Switzerland). Both sheath and auxiliary gases were nitrogen. The temperature of the vaporizer was 400 °C; the flow rate of acetonitrile was 350 μL min^−1^. The heated capillary temperature was 150 °C; the corona discharge current was 5 μA. Samples were dissolved in acetonitrile and introduced into the ion source through a Reodyne injector with a 5 μL loop. The program Xcalibur, version 1.3, was used for the data collection and treatment.

The molecular structure of compound **7** was confirmed by X-ray structure determination from powder data measured at room temperature on a diffractometer Huber G670 Guinier camera (Cu K_α__1_ radiation, λ = 1.54059 Å) equipped with an imaging-plate detector. The powder pattern was indexed in the monoclinic unit cell parameters, and the crystal structure was solved with the use of simulated annealing technique [45] and refined with the program MRIA [46] following the known procedures described by us earlier [47,48]. The crystal data, data collection, and refinement parameters are given in Table 1. The molecular structure of **7** and a portion of the crystal packing prepared with mercury [40] are shown in Figure 12 and in Figure S6 of the SM, respectively.

General procedure. A solution of organotrichlorosilane (**1** or **2,** or **3**) in acetone was added at 2–4 °C under stirring to a mixture of water and ice (1:1) taken in an amount that was necessary to get the required C_HCl_ after the complete hydrolysis of the organotrichlorosilane. The mixture was then placed into a fridge operated at 4 °C. Products precipitated from the solution in time. The precipitates were isolated at several time periods. Each precipitate was dissolved in diethyl ether; the ether layer was separated, washed with water until the neutral reaction of washing water, and dried over Na_2_SO_4_. The solvent was then removed and the yield of the product mixture was determined by the equation: *S_p_* = *100m_p_M_cl_/m_cl_*(*M_h_ − 18*), where *S_p_* is the yield of precipitate in percent; *m_p_*—the mass of the precipitates deposited up to and including the specified time point; *M_cl_*—the molecular mass of the corresponding organotriclorosilane; *m_cl_*—its mass taken for the reaction; *M_h_*—the molecular mass of its hydrolysate; and *18*—the molecular mass of water (see Note 1 in the SM and Figure 1, Figure 7 and Figure 9). The compositions of the precipitates were analyzed by APCI-MS, ^1^H, and ^29^Si NMR.

*Synthesis of all cis-(tetrahydroxy)(tetra-m-tolyl)cyclotetrasiloxane (4).* A solution of *m*-tolyltrichlorosilane (**1**, 2.17g, 9.6 mmol) in 58 mL of acetone was added to a mixture of water and ice (1:1, 480 g). The resulting reaction mixture was stirred at 2–4 °C for 15 min (the C_HCl_ achieved a value of 0.054 mol L^−1^ because of the hydrolysis). The procedure followed was as above. After 475 h, the combined white precipitate (0.64 g, 43.8% yield) was obtained. Recrystallization from diethyl ether gave (0.45 g, 30.8%) of **4** with m.p. = 160 °C, R_f_ = 0.03 (TLC, eluent: diethylether:toluene = 1:1). ^1^H NMR (600 MHz, acetone-d_6_, δ/ppm): 2.14 (s, 3H, CH_3_), 6.21 (s, 1H, OH), 7.12 (t, *J* = 6.0 Hz, 1H), 7.15 (d, *J* = 12.0 Hz, 1H), 7.28 (s, 1H), 7.36 (d, *J* = 6.0 Hz, 1H); ^29^Si NMR (acetone-d_6_, δ/ppm): −70.16; PI APCI-MS, *m*/*z*: 626 ([M+H_2_O]^+•^). The spectra coincided with those of **4** previously obtained by us. The same was valid for the R_f_ and m.p. values [37]. Elemental analysis: calculated (%) for C_28_H_32_Si_4_O_8_: C, 55.23; H, 5.30; Si, 18.45. Found: C, 55.33; H, 5.32; Si, 18.45.

*Synthesis of isomers of (tetrahydroxy)(tetra-m-chlorophenyl)cyclotetrasiloxane (6a and 6b).* The reaction was carried out in a similar fashion with a solution of *m*-chlorophenyltrichlorosilane (**2**) (**2**, 6.05 g, 25 mmol) in 171 mL of acetone and a mixture of water and ice (1160 g) but with stirring for 1 h (C_HCl_ was 0.056 mol L^−1^). The combined white precipitate (2.89 g, 68.5% yield) was collected after 720 h. It was washed with benzene to give 2.25 g (53.3%). Fractional recrystallization from a diethyl ether-hexane solution furnished 1.05 g (24.9% yield) of isomer **6a** with m.p. > 350 °C and R_f_ = 0.29 (TLC, eluent: diethylether:toluene = 2:0.5) and 0.80 g (19.0%) of isomer **6b** with m.p. > 250 °C and R_f_ = 0.05. Isomer **6a:** ^1^H NMR (400 MHz, acetone-d_6_, δ/ppm): 6.70 (s, 1H, OH), 7.38 (t, *J* = 8.0 Hz, 1H), 7.46 (d, *J* = 8.0 Hz, 1H), 7.67 (d, *J* = 8.0 Hz, 2H); ^29^Si NMR (acetone-d_6_, δ/ppm): −71.83. The spectrum and m.p. were the same as in the case of **6a** synthesized previously [37]. Elemental analysis: calculated (%) for C_24_H_20_Si_4_Cl_4_O_8_: C, 41.74; H, 2.91; Si, 16.27; Cl, 20.53. Found: C, 42.10; H, 2.98; Si, 16.24; Cl, 20.20. Isomer **6b:** ^1^H NMR (400 MHz, acetone-d_6_, δ/ppm): 6.74 (s, 1H, OH), 7.28 (t, *J* = 8.0 Hz, 1H), 7.43 (d, *J* = 8.0 Hz, 2H), 7.66 (d, *J* = 8.0 Hz, 1H); ^29^Si NMR (acetone-d, δ/ppm): −71.55. Elemental analysis: calculated (%, see for **6a**). Found: C, 42.10; H, 2.98; Si, 15.93; Cl, 20.19.

*Synthesis of 1,1,3,3-tetrahydroxy-1,3-α-dinaphtyldisiloxane.* As above for **2** with a solution of *a*-naphtyltrichlorosilane (**3**, 1.25 g, 4.78 mmol) in 14 mL of acetone and 80 g of water–ice mixture (C_HCl_ achieved a value of 0.15 mol L^−1^). The precipitates were taken after 24, 48, and 192 h. They were treated analogously to the above procedure for **2** to afford 0.63 g of reaction products. Each of them recrystallized from acetonitrile produced isomer **7** as a white powder (0.45g, 48% common yield) with m.p. 162–164 °C. ^1^H NMR (600 MHz, CD_3_CN, δ/ppm): 4.81 (s, 2H, OH), 7.43(t, *J* = 9.0 Hz, 1H), 7.47 (t, *J* = 6.0 Hz, 1H), 7.50 (t, *J* = 6.0 Hz, 1H), 7.89 (d, *J* = 6.0 Hz, 1H), 7.95 (d, *J* = 6.0 Hz, 1H), 7.98 (d, *J* = 6.0 Hz, 1H), and 8.36 (d, *J* = 12.0 Hz 1H); ^29^Si NMR (CD_3_CN, δ/ppm): −61.51. For X-ray powder diffraction data, see Table 1 and Table 2, Figure 12, and Figures S6 and S7 in the SM.

## 4. Conclusions

The results obtained showed that the acidity of the solutions affected the rate of the hydrolysis–condensation reaction of organotriclorosilanes and the yields of the products, and also, which products formed. In the case of *m*-tolyltrichlorosilane (**1**), e.g., all *cis*-(tetrahydroxy)(tetra-*m*-tolyl)tetracyclosiloxane (**4**) was obtained as the main final product when the reaction was conducted at C_HCl_ = 0.054 mol L^−1^ for 475 h. Meanwhile, a mixture containing many *m*-tolylcyclosiloxanes was separated from the 24 h reaction mixture, but APCI-MS showed two main peak groups corresponding to **4** and *m*-tolylcyclosiloxanes **5a** and/or **5b** when the reaction was carried out at C_HCl_ = 0.40 mol L^−1^ for 48 and more hours.

When *α*-naphtyltriclorosilane (**3**) was reacted with C_HCl_ = 0. 15 mol L^−1^ for 192 h, recrystallization of the combined precipitates obtained at 24, 48, and 192 time points from acetonitrile gave *trans*-isomer of 1,1,3,3-tetrahydroxy-1,3-di-*α*-naphtyldisiloxane (**7**) which was characterized by ^1^H and ^29^Si-NMR spectroscopy, and X-ray powder diffraction. The conformers of this compound should be the products of the first act of the condensation, and they were detected by PI APCI-MS as a peak at *m*/*z* 391 in the precipitates obtained in the cases of the reactions implemented at all values of the acidity employed. However, the corresponding disiloxanes were not found in the case of *m*-tolyltrichlorosilane (1) since they seemed to be more soluble than **7** and its stereoisomers, and thus, rapidly entered into the following condensation. As aforesaid, tetracyclosiloxane **4** was isolated instead.

The difference in products obtained at different acidities of the solutions was also observed in the case of **3**. Thus, in the PI APCI mass spectrum obtained for the precipitates collected after 48 and 240 h when the reaction was carried out at C_HCl_ = 0.037 mol L^−1^, two main ion peak groups were found corresponding to the monohydrates of *α*-naphtyl-T_8_ and *α*-naphtyl-T_10_, whereas in the case of C_HCl_ = 0.15 mol L^−1^ after 192 h, the most abundant peaks turned out to be the ion peaks of four or five compounds [*α*-naphtyl(HO)_2_SiO]_2_(*α*-naphtyl)(HO)Si and possibly, [*α*-naphtylSi(OH)O]_3_, [*α*-naphtylSi(OH)O]_4,5_, and *α*-naphtyl-T_8_.

In summary, it should be pointed out that the use of both ^29^Si NMR and APCI-MS proved to be a good tool for monitoring the hydrolysis–condensation reaction studied. We believe that it can be used for the investigation of other similar reactions.

A new compound synthesized, *trans*-1,1,3,3-tetrahydroxy-1,3-di-*α*-naphtyldisiloxane (**7**) can be a block for the obtainment of poly-*α*-naphtylsilsesquioxanes of different structures.

## Figures and Tables

**Table 1:** Crystal data for **7**.

Empirical Formula	C_20_H_18_O_5_Si_2_
M_r_	394.52
Crystal system	Monoclinic
Space group	*P2_1_/c*
Wavelength, Å	1.54059
Unit cell dimensions	
*a*, Å	13.5727(12)
*b*, Å	8.4084(7)
*c*, Å	8.2589(6)
*β,* ^o^	96.880(11)
Volume, Å^3^	935.76(13)
Z	2
D_x_ (Mg m^−3^)	1.400
μ, mm^−1^	1. 981
2θ_min_–2θ_max_, ∆2θ (^o^)	3.00–90.00, 0.01
No. parameters/restraints	327/402
R_p_, R_wp_, R_exp_	0.0331, 0.0420, 0.0193

**Table 2:** Hydrogen-bonding geometry (Å, °) for **7**.

*D−H…A*	*D−H*	*H…A*	*D…A*	*D−H…A*
O2−H2…O3^i^	0.82	1.89	2.674(5)	160
O3−H3…O2^ii^	0.82	2.24	2.759(5)	122

Symmetry codes: (i) *x*, 3/2-*y*, –1/2+*z*; (ii) 1-*x*, –1/2+*y*, 3/2-*z*.

## Supplementary Materials

The following are available online. Notes 1–7. Figure S1: PI APCI mass spectrum of products from the hydrolysis–condensation reaction of compound **1** carried out in a water-acetone solution at C_HCl_ = 0.40 mol L^−1^ for 120 h. Figure S2: PI APCI mass spectrum of products from the hydrolysis–condensation reaction of **1** carried out in a water-acetone solution at C_HCl_ = 0.15 mol L^−1^ for 48 h. Figure S3: ^29^Si NMR spectra of the precipitates deposited from the reaction mixtures of the hydrolysis–condensation reaction of **2** conducted at 4 °C: (**a**) at C_HCl_ = 0.032 mol L^−1^ for 90 h and (**b**) at C_HCl_ = 0.056 mol L^−1^ for 72 h. Figure S4: PI APCI mass spectra of products from the hydrolysis-condensation reaction of α-naphtyltrichlorosilane (**3**) carried out in water-acetone solutions with C_HCl_ = 0.037 mol L−1 at 4 °C for: (a) 48 h and (b) 240 h. Figure S5: PI APCI mass spectrum of the precipitate collected from the reaction of species **3** conducted at 4 °C and C_HCl_ = 0.15 mol L^−1^ for 192 h. Figure S6: A portion of the crystal packing of **7** viewed approximately along [011] and showing the hydrogen-bonded (thin blue lines) layers of the molecules with the naphthalene bicycles protruding in the opposite directions. Figure S7: The final Rietveld plot for **7**. The experimental and difference (experimental minus calculated) diffraction profiles are shown as the black and red lines, respectively. The vertical blue bars correspond to the calculated positions of the Bragg peaks. The structure of compound **7** was registered in the database of the Cambridge Crystallographic Data Centre (CCDC) (http://www.ccdc.cam.ac.uk.”) under the number 1867122. The data can be obtained free of charge from it.
