# Supplementary material for: A Study of the Influence of the HCl Concentration on the Composition and Structure of (Hydroxy)Arylsiloxanes from the Hydrolysis–Condensation Reaction of Aryltrichlorosilanes
Source: Molecules. 2019 Nov 19;24(22):4195. doi: 10.3390/molecules24224195 (PMC6891358; doi:10.3390/molecules24224195)
Supplement: Supplementary file 1 [file molecules-24-04195-s001.pdf]

Supplementary Materials

A study of the influence of the HCl concentration on the composition and structure of (hydroxy)arylsiloxanes from the hydrolysis–condensation reaction of aryltrichlorosilanes

Irina M. Petrova,[1] Yury I. Lyakhovetsky,[1] Vladimir V. Chernyshev,[2,3] Nikolai S. Ikonnikov,[1] and Nataliya N. Makarova.[1,*]

[1] A. N. Nesmeyanov Institute of Organoelement Compounds of the Russian Academy of Sciences, 28 Vavilov St., 119991 Moscow V-334, Russia, nmakar@ineos.ac.ru

[2] Department of Chemistry of the M.V. Lomonosov Moscow State University, GSP-1, 1 Leninskie Gory, 119991 Moscow, Russia; vladimir@struct.chem.msu.ru

[3] A. N. Frumkin Institute of Physical Chemistry and Electrochemistry of the Russian Academy of Sciences, 31 Leninsky prospect, Moscow 119071, Russian Federation.

*****Correspondence: nmakar@ineos.ac.ru; Tel.: +7-499-135-92-43 (N.N.M.)

Notes

Note 1. The relatively low temperature (4°C) was employed for the experiments since it allowed careful monitoring of the reactions by a combination of APCI-MS and ^{29}Si NMR. This gave possibility to detect intermediates that was important for elucidation of the reaction mechanism. Moreover, HCl could catalyze both the formation and the rapture of SI-O-Si bonds and thus, the reaction most likely involved equilibriums. Since the condensations examined appeared to be exothermic, higher temperatures could have shifted the equilibriums towards compounds with less degree of condensation. So that, the study of the temperature influence on the reactions is a matter of a separate and thorough investigation.

The concentrations of the reagents were connected with the HCl concentrations by a factor of 3 since the acid formed because of the hydrolysis organotrichlorosilanes. The maximal concentration C_{HCl} = 0.40 mol L^{-1} was chosen since at this concentration the reaction of **1** was already completed for 120 h and this provided the possibility to obtain analytical data at the earlier points (24 and 48 h) and elucidate a lot of intermediates. All other acidities employed were obtained by the respective dilution of the reaction mixture.

Note 2. The yields of the products given in Figures 1, 7, and 9 were calculated on the assumption that each molecule of organotrichlorosilane changed three chlorine atoms for three hydroxyl groups and every molecule with hydroxyls formed lose a water molecule in the course of condensation. Certainly, the latter was valid in the cases when organocyclosiloxanes with one

OH-group at each silicon atom were formed. However, the errors were insignificant for all other organosiloxane products.

Note 3. One cannot exclude certain contribution to the intensities of the signals from compounds molecules of which contained the silicon atoms of different types.

Note 4. One cannot tell whether the hydrate was formed upon addition of a water molecule in the mass spectrometer to the radical cation of **4** (or to any other compound), or the hydrolysis occurred to give an ion with two hydroxyl groups. For the simplicity, the first variant is depicted in the figures of the mass spectra.

Note 5. Certainly, mass spectrometer is a chemical reactor and the reaction of analytes can occur during the analysis procedures. In the case of the APCI variant equipped with an ion trap mass analyzer, the bimolecular and ion/molecule reactions can proceed in the vaporizer or the ion source and the latter reactions in the mass analyzer. However, the mass spectrum of isolated compound 4 showed other ion peaks to be of negligible abundances. This fact contradicts the possibility that the reactions of the hydrolysate of **1** occurred inside the mass spectrometer rather than outside of it in a flask.

Another point should also be mentioned here. The mass numbers of ions in the mass spectra of product mixtures couldn't certainly provide complete identification of the products, especially since one or two water molecule could add to some ions. However, with a great degree of probability the structures depicted in the mass spectral figures seem to be valid.

Note 6. In those cases when the peak abundances of the nominal mass ions were noticeably less than those calculated for the corresponding ion groups, the protonated molecules seemed to contribute to the abundances.

Note 7. The attempts of monitoring the reactions of organotrichlorosilane **2** by PI APCI-MS failed since we couldn't record the mass spectra of the samples. The same occurred for the attempt to obtain the EI mass spectrum of product **6a**, most likely, because of its low volatility.

Note 8. As in the case of compound **1** (Note 5), the ions registered in the mass spectra were due to the products formed in the reactions occurring in flasks rather than in the mass spectrometer. Actually, the dynamics in the ion abundances was observed when the analyses were performed in time, the abundance of the peaks of **7** strongly decreasing, whereas those of other products increasing. This finding does not correlate with the possibility that the reactions proceeded in the mass spectrometer.

Additional mass and ^{29}Si NMR spectra

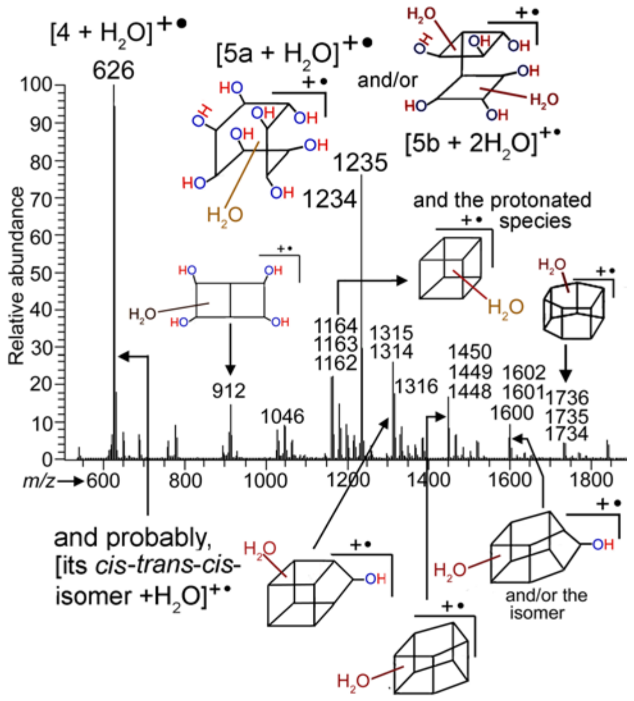

Figure S1. PI APCI mass spectrum of products from the hydrolysis-condensation reaction of compound **1** carried out in a water-acetone solution at C_{HCl} = 0.40 mol L^{-1} for 120 h.

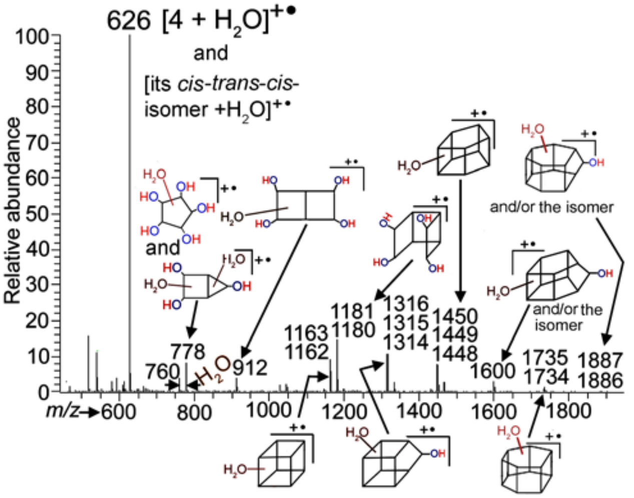

Figure S2. PI APCI mass spectrum of products from the hydrolysis-condensation reaction of **1** carried out in a water-acetone solution at C_{HCl} = 0.15 mol L^{-1} for 48 h.

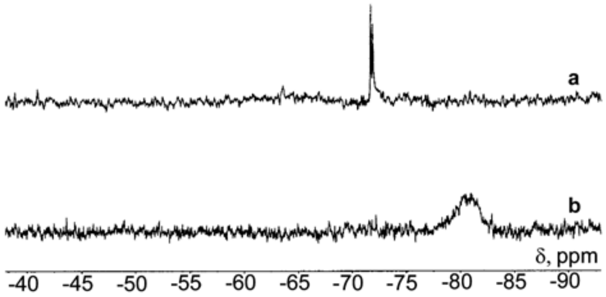

Figure S3. ^{29}Si NMR spectra of the precipitates deposited from the reaction mixtures of the hydrolysis-condensation reaction of **2** conducted at 4°C: **a** – at C_{HCl} = 0.032 mol L^{-1} for 90 h and **b** – at C_{HCl} = 0.056 mol L^{-1} for 72 h.

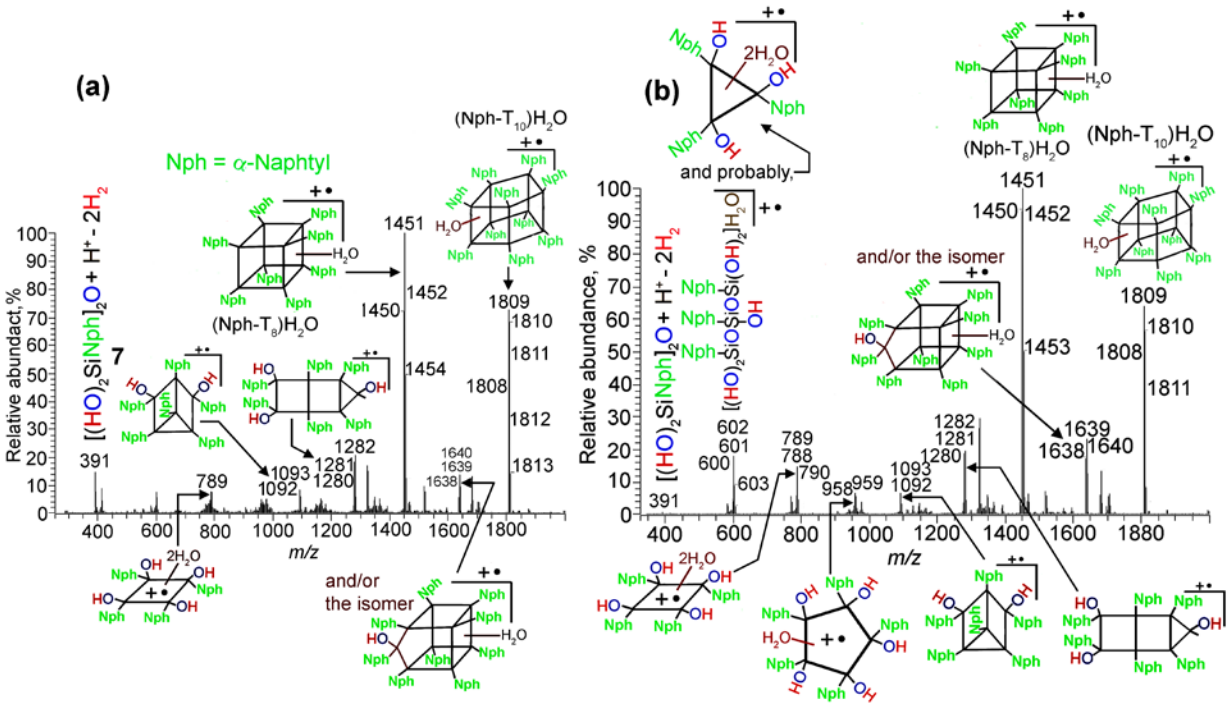

Figure S4. PI APCI mass spectra of products from the hydrolysis-condensation reaction of α-naphtyltrichlorosilane (**3**) carried out in water-acetone solutions with C_{HCl} = 0.037 mol L^{-1} at 4 °C for: **a)** 48 h and **b)** 240 h.

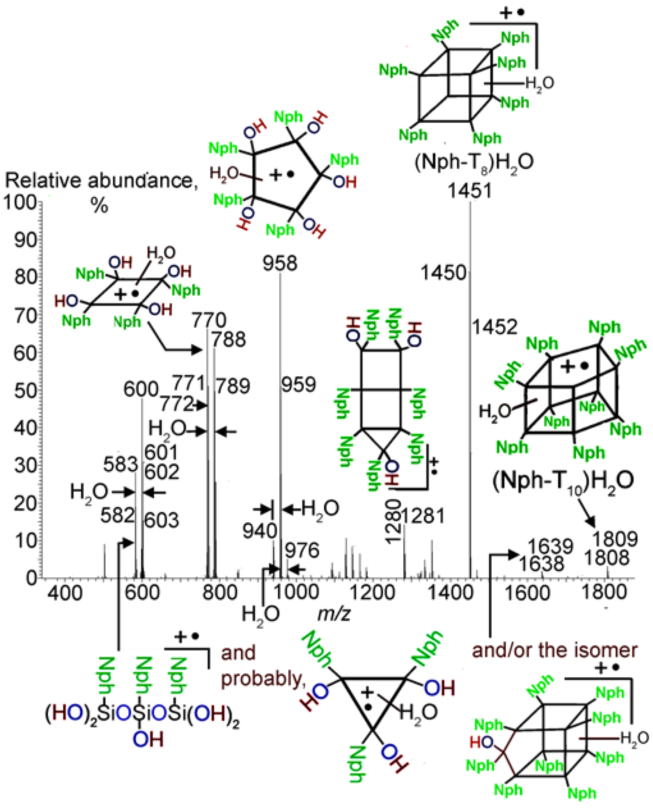

Figure S5. PI APCI mass spectrum of the precipitate collected from the reaction of species **3** conducted at 4 °C and C_{HCl} = 0.15 mol L^{-1} at a time point of 192 h.

Additional X-ray powder diffraction analysis data

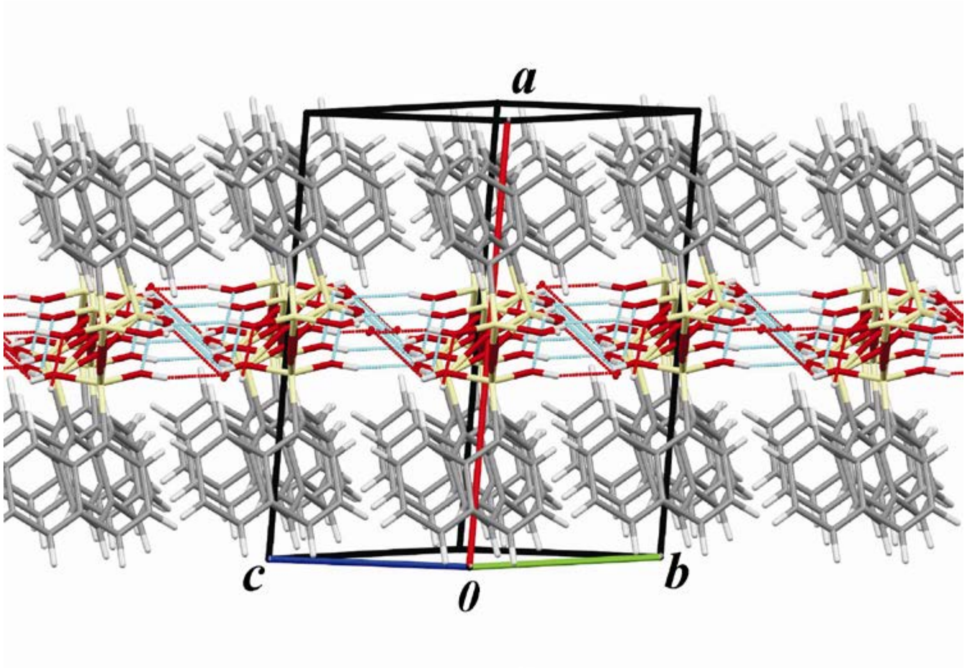

Figure S6. A portion of the crystal packing of **7** viewed approximately along [011] and showing the hydrogen-bonded (thin blue lines) layers of the molecules with the naphthalene bicycles protruding in the opposite directions.

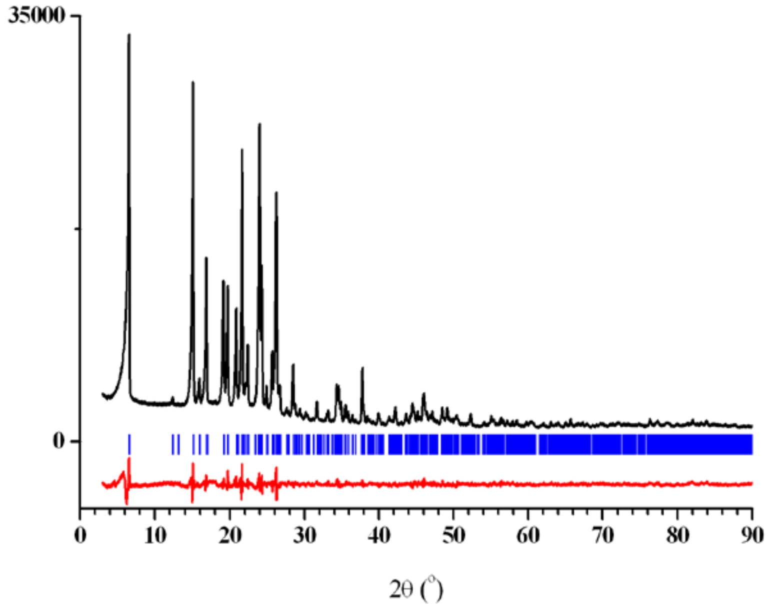

Figure S7. The final Rietveld plot for **7**. The experimental and difference (experimental minus calculated) diffraction profiles are shown as the black and red lines, respectively. The vertical blue bars correspond to the calculated positions of the Bragg peaks.
